# Machine learning insights into early mortality risks for small cell lung cancer patients post-chemotherapy

**DOI:** 10.3389/fmed.2025.1483097

**Published:** 2025-01-24

**Authors:** Min Liang, Fuyuan Luo

**Affiliations:** ^1^Department of Respiratory and Critical Care Medicine, Maoming People’s Hospital, Maoming, China; ^2^Center of Respiratory Research, Maoming People’s Hospital, Maoming, China; ^3^Department of Respiratory and Critical Care Medicine, Gaozhou People's Hospital, Maoming, China

**Keywords:** small cell lung cancer, early mortality, machine learning, survival, chemotherapy

## Abstract

**Introduction:**

Small cell lung cancer (SCLC) is a highly aggressive form of lung cancer, and chemotherapy remains a cornerstone of its management. However, the treatment is associated with significant risks, including heightened toxicity and early mortality. This study aimed to quantify the 90-day mortality rate post-chemotherapy in SCLC patients, identify associated features, and develop a predictive machine learning model.

**Methods:**

This study utilized data from the Surveillance, Epidemiology, and End Results (SEER) database (2000–2018) to identify prognostic features influencing early mortality in SCLC patients. Prognostic features were selected through univariate logistic regression and Lasso analyses. Predictive modeling was performed using advanced machine learning algorithms, including XGBoost, Multilayer Perceptron, K-Nearest Neighbor, and Random Forest. Additionally, traditional models, such as logistic regression and AJCC staging, were employed for comparison. Model performance was evaluated using key metrics, including the Area Under the Receiver Operating Characteristic Curve (AUC), calibration plots, the Kolmogorov–Smirnov (KS) statistic, and Decision Curve Analysis (DCA).

**Results:**

Analysis of 12,500 eligible patients revealed 10 clinical features significantly impacting outcomes. The XGBoost model demonstrated superior discriminatory capability, achieving AUC scores of 0.95 in the training set and 0.78 in the validation set. It outperformed comparative models across all datasets, as evidenced by its AUC, KS score, calibration, and DCA results. Additionally, the model was integrated into a web-based platform to improve accessibility.

**Conclusion:**

This study introduces a machine learning model alongside a web-based support system as critical resources for healthcare professionals, facilitating personalized clinical decision-making and enhancing treatment strategies for SCLC patients post-chemotherapy.

## Introduction

Lung cancer ranks as the foremost cancer type worldwide and remains the principal cause of death (1). Small cell lung cancer (SCLC), representing about 10–15% of all lung cancer pathologies, is notorious for its aggressive nature, low degree of differentiation, rapid advancement, and bleak outcomes (2). Research reveals a stark prognosis for patients with Extensive-Stage Small Cell Lung Cancer (ES-SCLC), where the median Overall Survival (OS) is alarmingly brief at roughly 10 months (3, 4), and the likelihood of survival at 5 years plummets to less than 5% (5).

The most prevalent genetic mutations in SCLC involve the inactivation of TP53 and RB1 genes. Despite this, targeted therapies for SCLC remain elusive (6). Immunotherapy has brought significant advancements to the treatment landscape, yet platinum-based chemotherapy, with or without radiotherapy, remains the standard first-line treatment. Absence of chemotherapy leads to a drastic decline in survival rates, underscoring SCLC’s aggressive nature and its initial responsiveness to the therapy (7). Consequently, chemotherapy is recommended even for elderly patients with poor Performance Status (PS) and significant comorbidities. Nevertheless, the occurrence of early mortality post-chemotherapy points to a notable treatment challenge.

Several studies have concentrated on understanding early mortality in SCLC, yet they often face limitations due to patient selection heterogeneity and variable model performance, especially when traditional modeling algorithms are used (8, 9). Recently, however, oncology modeling has seen significant advancements with the integration of machine learning. These computational techniques have demonstrated remarkable accuracy in predicting cancer progression and treatment responses (10). While existing prediction models primarily rely on machine learning to assess OS in post-chemotherapy SCLC patients, often using tree-based and radiomic approaches (11, 12), there remains a gap in models developed with other types of machine learning algorithms, particularly for predicting early mortality.

This study harnesses the Surveillance, Epidemiology, and End Results (SEER) database to forge prognostic models employing machine learning techniques aimed at predicting early mortality among SCLC patients receiving chemotherapy. It conducts an exhaustive comparative analysis to assess the efficacy of these models against conventional logistic regression and AJCC staging systems. The outcomes of this research have culminated in the development of an accessible, web-based classifier that provides visual insights, establishing itself as an indispensable asset for informed clinical decision-making.

## Methods

### Raw data source

Managed by the National Cancer Institute (NCI), the SEER program serves as an extensive repository of cancer statistics in the United States. It compiles and disseminates data on cancer incidence and survival from registries that span approximately 34.6% of the U.S. population. The study accessed this dataset after fulfilling the SEER Research Data Agreement and extracted clinicopathological details using the SEER*Stat software version 8.4.0.1.^1^

### Patients and study endpoint

This study included patients diagnosed with SCLC between 2000 and 2018, as defined by the International Classification of Diseases for Oncology, Third Edition (ICD-O-3), with site codes C34.0-C34.9 and histological type code 8041. Eligibility for the study required a pathologically confirmed diagnosis of SCLC, treatment with chemotherapy, and the presence of a single primary tumor without other malignancies recorded in the database.

Patients were excluded if they lacked detailed demographic information or comprehensive clinicopathological data, including the primary tumor’s site, size, laterality, histologic grade, and American Joint Committee on Cancer (AJCC) stage. Additionally, individuals were omitted if records of chemotherapy or radiotherapy treatment were incomplete or if survival status and follow-up data were missing.

The study’s primary endpoint, early mortality, was specifically identified as death within 90 days post-diagnosis among those who received chemotherapy, with those surviving beyond 90 days serving as the comparison group.

### Baseline characteristics presentation

Initially, the clinical and demographic characteristics of the study population were methodically outlined. Continuous variables were summarized by their mean and standard deviation, while categorical variables were described via frequencies and percentages. The detailed analysis included 17 features aimed at identifying independent prognostic features in patients with SCLC. Demographic aspects such as age, sex, race, and marital status, as well as clinicopathological features of the tumor, including site, size, laterality, grade, and AJCC stage, were carefully evaluated. Furthermore, relevant treatment information, including chemotherapy and radiotherapy, was explored.

### Feature engineering and data balancing

In the process of feature engineering and addressing data imbalance, the study initially applied Spearman correlation analysis to explore the relationships among data features. This method assesses monotonic relationships, shedding light on potential associations within the dataset and facilitating the identification of patterns through the creation of a correlation heat map.

Subsequently, to enhance machine learning model performance, the study implemented categorical label encoding, transforming categorical variables into binary matrices that represent category memberships through one-hot encoding. The challenge of class imbalance in outcome status was tackled by employing the Synthetic Minority Over-sampling Technique (SMOTE). SMOTE addresses this issue by generating synthetic samples from the minority class. It selects a random point from the minority class, identifies its k-nearest neighbors, and creates synthetic instances between the chosen point and its neighbors. This method achieves a more balanced class distribution and improves the model’s ability to recognize patterns associated with the minority class, thereby enhancing prediction accuracy for these cases.

### Model construction and validation approach

Eligible patients were methodically divided into training and validation datasets in a 7:3 ratio through random allocation, providing a robust foundation for analysis. The training dataset played a crucial role in the development of the prognostic model and risk assessment classification, significantly refining the analytical approach. The validation dataset, maintained separately from its training counterpart, was essential in evaluating the model’s performance and ensuring the reliability of the findings. Within the prognostic framework, the classification threshold was optimized using 10-fold cross-validation on the training set, with the goal of maximizing the Area Under the Receiver Operating Characteristic Curve (AUC) to enhance the model’s predictive precision and generalizability.

To bolster the reliability and robustness of the model, the process began by screening potential prognostic features using univariate logistic regression. Following this preliminary analysis, the Lasso model was employed to pinpoint essential prognostic features. This two-step approach combines the strengths of the logistic proportional hazards model with the Lasso technique, effectively isolating significant features while minimizing the influence of less relevant ones. Subsequently, traditional predictive models were developed using logistic regression, incorporating the key determinants unearthed by the Lasso model. In parallel, the AJCC staging model was constructed using its distinct criteria. To identify the most accurate predictive tool, machine learning models, including XGBoost, Multilayer Perceptron (MLP), K-Nearest Neighbor (KNN), and Random Forest (RF), were trained and analyzed using the tidymodels package in R. Bayesian optimization was employed to fine-tune the hyperparameters of these models, seamlessly integrating this technique into the machine learning workflow. The primary objective was to determine the model that most effectively predicts OS rates, thereby establishing an optimal prognostic framework.

XGBoost, a gradient boosting algorithm, was configured with 300 trees and a stopping criterion of 25 iterations without improvement, with a 20% validation split for model evaluation. The model’s hyperparameters, including mtry, min_n, tree depth, learning rate, loss reduction, and sample size, were optimized using Bayesian optimization. The workflow integrated a preprocessing recipe and the tuned model for streamlined analysis. Hyperparameter tuning was performed through 50 iterations with 15 initial configurations, guided by classification performance metrics, with optimization controlled for no improvement over 15 iterations.

The MLP, implemented via the nnet engine, was optimized for hidden units, regularization penalty, and epochs. A maximum weight parameter of 10,000 ensured scalability for larger networks. The analysis combined data preprocessing with model training in a streamlined workflow.

The KNN, configured with the kknn engine, optimized hyperparameters such as neighbors (neighbors, 5–35) and weighting functions. The distance metric was fixed at a power of 2. The workflow seamlessly integrated data preprocessing and the tuned model for classification.

The RF model, implemented using the randomForest engine, was optimized by tuning key parameters, including the mtry from 2 to 15, the total number of trees (ranging from 100 to 3,000), and the min_n from 7 to 55. Feature importance was assessed to evaluate the contributions of each variable. A streamlined workflow was established to efficiently integrate preprocessing and model training, ensuring a smooth and systematic analysis process.

### Model performance evaluation

The performance of the model underwent a detailed evaluation using a set of well-established metrics. This comprehensive analysis included Receiver Operating Characteristic (ROC) curve analysis, calibration curve analysis, Decision Curve Analysis (DCA), and Kolmogorov–Smirnov (KS) statistic. The ROC curve, by quantifying the AUC, provided a crucial measure of the model’s ability to distinguish between outcomes, serving as a cornerstone of prognostic accuracy. DCA offered insights into the model’s clinical utility by evaluating the net benefits at different probability thresholds. Calibration analysis examined how well the model’s predicted probabilities matched actual outcomes, aiming for a model that closely aligns with the 45° diagonal in the calibration plot. The KS statistic is a valuable measure for comparing the cumulative distribution functions of two or more samples. In the context of machine learning and model evaluation, a higher KS statistic indicates a greater separation between the distributions of the positive and negative classes predicted by the model.

### Model interpretation

The study utilized SHAP (SHapley Additive exPlanations) to interpret the machine learning model, clarifying the contribution of each variable and fostering greater model transparency. SHAP identifies influential features, enhancing the understanding of model predictions—an essential aspect for informed decision-making and model enhancement. The beeswarm summary plot, a feature of SHAP, graphically displays the impact of variables, providing a comprehensive view of their influence on the model’s results. This method enables practitioners to pinpoint the main features that shape model predictions, ensuring reliable and credible outcomes in complex machine learning endeavors.

To enhance the understanding of how features influence model performance, both breakdown and partial dependence analyses were employed. Breakdown analysis provides an interpretive lens, dissecting a model’s prediction for a specific instance to reveal the contributions of each input feature. This technique is particularly insightful for understanding the role of individual features in shaping the model’s predictions on a case-by-case basis. It proves especially useful for complex models, granting detailed insight into the decision-making process at an individual level.

In contrast, partial dependence analysis assesses the impact of key features on the model’s predicted outcome by neutralizing the effects of all other features. This method is particularly valuable for understanding the intricacies of complex models, such as ensemble methods or neural networks, where the relationship between inputs and outputs can be intricate and not immediately clear. Partial Dependence Plots (PDPs) are utilized in this analysis to isolate and examine the influence of specific features. These plots provide insights into how the model responds across various values for those features, clarifying how certain features independently drive predictions, regardless of their interactions with other variables in the dataset.

### Statistical analysis

Statistical analyses in this study were conducted using R software (version 4.2.1^2^), employing two-tailed tests with a significance threshold of *p* < 0.05. The model was developed by integrating a suite of R packages such as “tidymodels,” “glmnet,” “dplyr,” “tidyr,” and “ggplot2.” The web-based dynamic model was constructed using the “shinydashboard” R package.

## Results

### Patient characteristics

**Figure 1:**
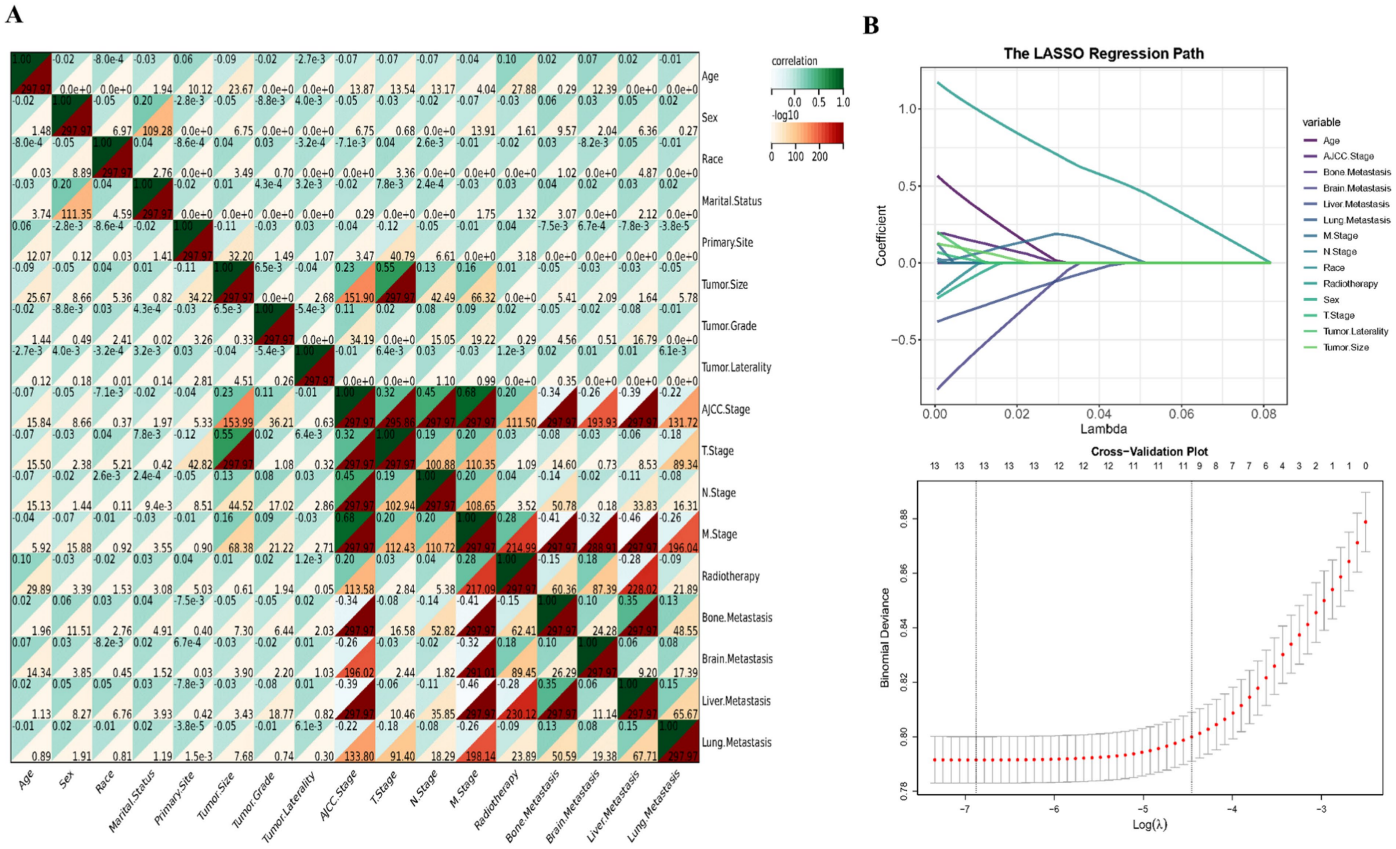
Procedure of feature screening. Correlation **(A)** and Lasso **(B)** analyses.

From an initial cohort of 23,102 patients diagnosed with SCLC who met the eligibility criteria, 12,500 individuals were selected for this study based on predefined inclusion parameters. Among the patients included, 19.03% experienced early mortality. The cohort predominantly comprised older individuals, with an average age exceeding 66 years. Within this group, the median observed mortality rate stood at 16%. Demographically, the majority were Caucasian (86.82%), with a slight majority being female (51.13%) and over half being married (52.31%). The primary tumors were most commonly located in the right side of the lung (57.48%) and the upper lung lobes (59.27%), with lower lobe occurrences at 23.71%. The cohort’s average tumor size was 52 millimeters, and advanced tumor stages accounted for the vast majority (89.73%) of cases. Metastases were observed in the bone (22.43%), brain (15.5%), liver (26.96%), and lung (11.98%). Radiotherapy was administered to 60.78% of the cohort. Table 1 and the ridge plot (Supplementary Figure S1) provide a comprehensive summary of the baseline characteristics. Spearman correlation analysis revealed minimal multicollinearity among the dataset’s features (Figure 1A).

**Table 1:** Baseline characteristics of SCLC patients post-chemotherapy.

Features	Total (*n* = 12,500)
Age(years), Mean ± SD	66.27 ± 9.44
Tumor size (mm), Mean ± SD	52.62 ± 38.11
**Sex, *n* (%)**	
Male	6,109 (48.87)
Female	6,391 (51.13)
**Race, *n* (%)**	
White	10,853 (86.82)
Black	1,131 (9.05)
Others	516 (4.13)
**Marital status, *n* (%)**	
Married	6,539 (52.31)
Unmarried	5,513 (44.10)
Unknown	448 (3.58)
**Primary tumor site, *n* (%)**	
Main bronchus	1,401 (11.21)
Upper lobe	7,409 (59.27)
Middle lobe	561 (4.49)
Lower lobe	2,964 (23.71)
Overlapped lesions	165 (1.32)
**Tumor grade, *n* (%)**	
Grade I	16 (0.13)
Grade II	27 (0.22)
Grade III	1,213 (9.70)
Grade IV	1949 (15.59)
Unknown	9,295 (74.36)
**Tumor laterality, *n* (%)**	
Left	5,304 (42.43)
Right	7,185 (57.48)
Bilateral	11 (0.09)
**T stage, *n* (%)**	
T1	2,043 (16.34)
T2	3,341 (26.73)
T3	2,836 (22.69)
T4	4,280 (34.24)
**N stage, *n* (%)**	
N0	2,017 (16.14)
N1	1,011 (8.09)
N2	6,885 (55.08)
N3	2,587 (20.70)
**M stage, *n* (%)**	
M0	4,897 (39.18)
M1	7,603 (60.82)
**AJCC stage**	
I	680 (5.44)
II	603 (4.82)
III	3,614 (28.91)
IV	7,603 (60.82)
**Bone metastasis, *n* (%)**	
Yes	2,804 (22.43)
No	9,561 (76.49)
Unknown	135 (1.08)
**Brain metastasis, *n* (%)**	
Yes	1,937 (15.50)
No	10,430 (83.44)
Unknown	133 (1.06)
**Liver metastasis, *n* (%)**	
Yes	3,370 (26.96)
No	9,007 (72.06)
Unknown	123 (0.98)
**Lung metastasis, *n* (%)**	
Yes	1,498 (11.98)
No	10,855 (86.84)
Unknown	147 (1.18)
**Radiotherapy, *n* (%)**	
Yes	7,598 (60.78)
None/unknown	4,902 (39.22)
**Status, *n* (%)**	
Alive	10,501 (84.01)
Dead	1,999 (15.99)

SD, Standard Deviation.

### Predictive feature identification and model development

The study undertook a thorough examination, analyzing a wide array of features including demographic details, tumor specifics, and treatment approaches, treating them as covariates in both univariate logistic regression and Lasso analyses. As depicted in Table 2, the initial univariate logistic regression analysis excluded marital status, primary tumor location, and tumor grade as standalone predictors of early mortality. The subsequent Lasso analysis refined feature selection, pinpointing 10 critical factors—age, sex, tumor size, tumor laterality, AJCC stage, T stage, M stage, radiotherapy, and metastases to the brain and liver—as key influencers of early mortality in SCLC patient. This selection process via the Lasso algorithm is illustrated in Figure 1B. Building on this foundation, the study developed a traditional prognostic model using logistic regression. To further enhance the accuracy of survival predictions, this study also crafted additional prognostic models based on the machine learning algorithms, aiming for a higher precision in forecasting patient outcomes.

**Table 2:** Univariate logistic regression analysis.

Features	S.E	Z-score	*p-*value	OR (95%CI)
Age	0.00	11.11	<0.001	1.04 (1.03 ~ 1.04)
Tumor size	0.00	4.97	<0.001	1.01 (1.01 ~ 1.01)
**Sex**				
Male				1.00 (Reference)
Female	0.06	−5.56	<0.001	0.72 (0.64 ~ 0.81)
**Race**				
White				1.00 (Reference)
Black	0.11	−2.91	0.004	0.72 (0.58 ~ 0.90)
Others	0.16	−1.68	0.092	0.77 (0.56 ~ 1.04)
**Marital status**				
Married				1.00 (Reference)
Unmarried	0.06	1.13	0.259	1.07 (0.95 ~ 1.20)
Unknown	0.15	1.90	0.058	1.33 (0.99 ~ 1.77)
**Tumor laterality**				
Left				1.00 (Reference)
Right	0.06	3.10	0.002	1.20 (1.07 ~ 1.35)
Bilateral	1.08	−0.02	0.982	0.98 (0.12 ~ 8.12)
**Primary site**				
Main bronchus				1.00 (Reference)
Upper lobe	0.09	−1.12	0.264	0.90 (0.75 ~ 1.08)
Middle lobe	0.16	−0.47	0.635	0.93 (0.67 ~ 1.27)
Lower lobe	0.10	−0.69	0.489	0.93 (0.76 ~ 1.14)
Overlapped lesions	0.25	0.42	0.671	1.11 (0.68 ~ 1.82)
**Tumor grade**				
Grade I				1.00 (Reference)
Grade II	1.47	−0.36	0.718	0.59 (0.03 ~ 10.48)
Grade III	1.05	0.44	0.661	1.59 (0.20 ~ 12.51)
Grade IV	1.05	0.55	0.581	1.79 (0.23 ~ 14.02)
Unknown	1.05	0.65	0.515	1.98 (0.25 ~ 15.47)
**AJCC stage**				
I				1.00 (Reference)
II	0.27	1.18	0.236	1.37 (0.81 ~ 2.31)
III	0.21	2.51	0.012	1.68 (1.12 ~ 2.51)
IV	0.20	7.34	<0.001	4.27 (2.90 ~ 6.28)
**T stage**				
T1				1.00 (Reference)
T2	0.10	3.55	<0.001	1.44 (1.18 ~ 1.77)
T3	0.10	4.83	<0.001	1.66 (1.35 ~ 2.03)
T4	0.10	6.42	<0.001	1.87 (1.55 ~ 2.27)
**N stage**				
N0				1.00 (Reference)
N1	0.14	−0.70	0.484	0.91 (0.68 ~ 1.20)
N2	0.09	4.04	<0.001	1.43 (1.20 ~ 1.70)
N3	0.10	3.58	<0.001	1.44 (1.18 ~ 1.75)
**M stage**				
M0				1.00 (Reference)
M1	0.07	14.62	<0.001	2.77 (2.42 ~ 3.17)
**Radiotherapy**				
Yes				1.00 (Reference)
None/unknown	0.06	20.85	<0.001	3.59 (3.18 ~ 4.04)
**Bone metastasis**				
Yes				1.00 (Reference)
No	0.06	−8.45	<0.001	0.58 (0.51 ~ 0.66)
Unknown	0.26	−0.22	0.826	0.94 (0.56 ~ 1.58)
**Brain metastasis**				
Yes				1.00 (Reference)
No	0.07	−7.47	<0.001	0.58 (0.50 ~ 0.67)
Unknown	0.27	0.20	0.839	1.06 (0.62 ~ 1.80)
**Liver metastasis**				
Yes				1.00 (Reference)
No	0.06	−15.30	<0.001	0.40 (0.35 ~ 0.44)
Unknown	0.28	−1.26	0.207	0.70 (0.40 ~ 1.22)
**Lung metastasis**				
Yes				1.00 (Reference)
No	0.08	−6.46	<0.001	0.60 (0.51 ~ 0.70)
Unknown	0.25	0.05	0.960	1.01 (0.62 ~ 1.65)

S.E., Standard Error; OR, Odds Ratio.

### Discriminatory ability and clinical utility of the predictive models

**Figure 2:**
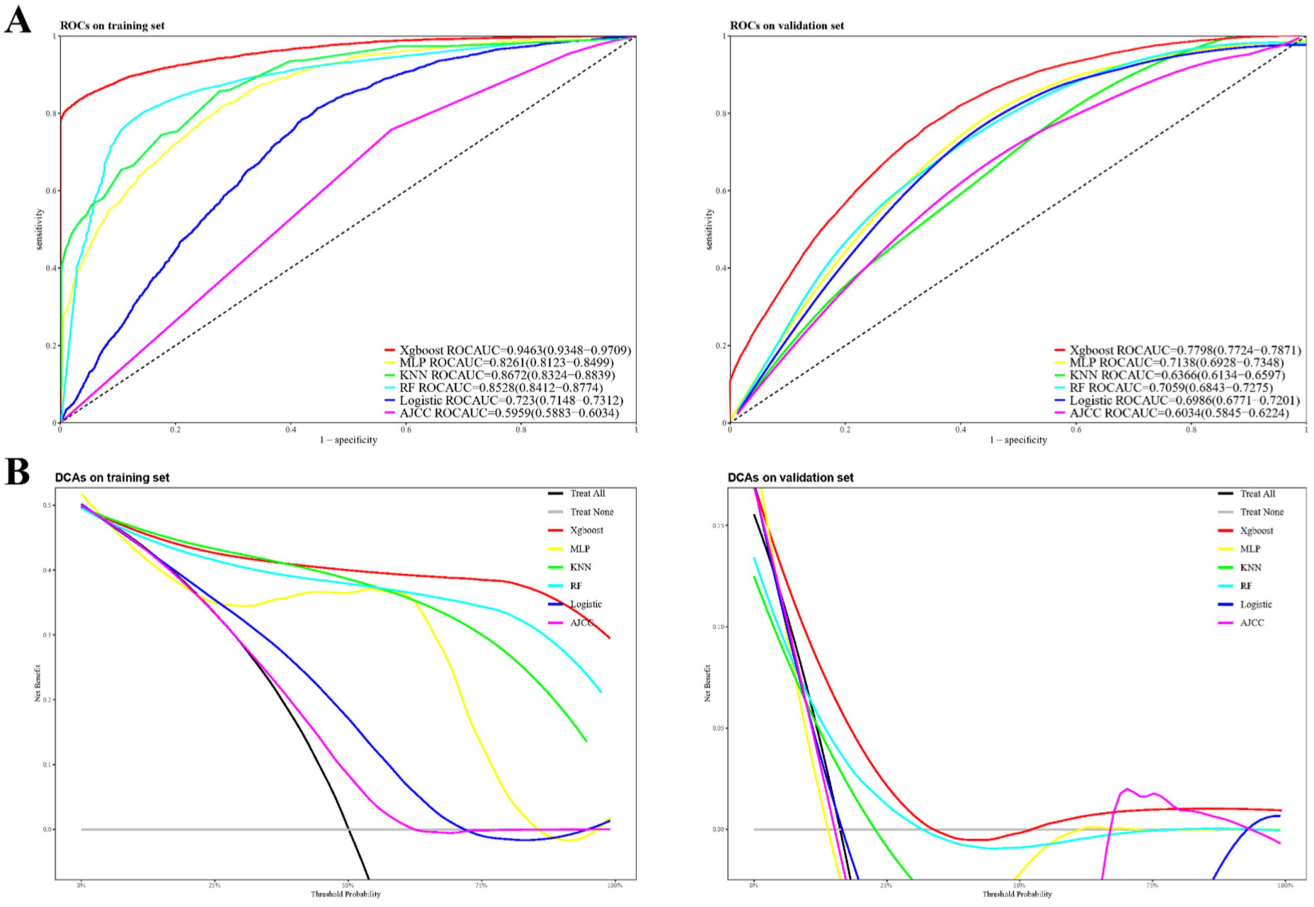
Model performance evaluation: AUCs **(A)** and DCAs **(B)** for training and validation datasets.

Figure 2A presents the performance metrics for the training and validation datasets. The logistic regression model achieved an AUC of 0.723 (95% CI 0.715–0.731) for early mortality prediction in the training set, which slightly declined to 0.699 (95% CI 0.677–0.72) in the validation set. The AJCC staging approach showed limited discriminatory power, with AUCs of 0.596 (95% CI 0.588–0.603) in the training set and 0.603 (95% CI 0.585–0.622) in the validation set.

In contrast, the XGBoost model demonstrated superior performance, with an AUC of 0.946 (95% CI 0.935–0.971) in the training set and 0.780 (95% CI 0.772–0.787) in the validation set. The MLP model attained AUCs of 0.826 (95% CI 0.812–0.850) in the training set and 0.714 (95% CI 0.693–0.735) in the validation set. Similarly, the KNN model achieved AUCs of 0.867 (95% CI 0.832–0.884) in the training set and 0.637 (95% CI 0.613–0.660) in the validation set, while the RF model recorded AUCs of 0.853 (95% CI 0.841–0.877) in the training set and 0.710 (95% CI 0.684–0.728) in the validation set. Figure 2B highlights the superior prognostic performance of the XGBoost model, as demonstrated by the DCA. This analysis shows that XGBoost outperforms both other machine learning algorithms and traditional approaches, such as logistic regression and AJCC staging, in both the training and validation datasets.

**Figure 3:**
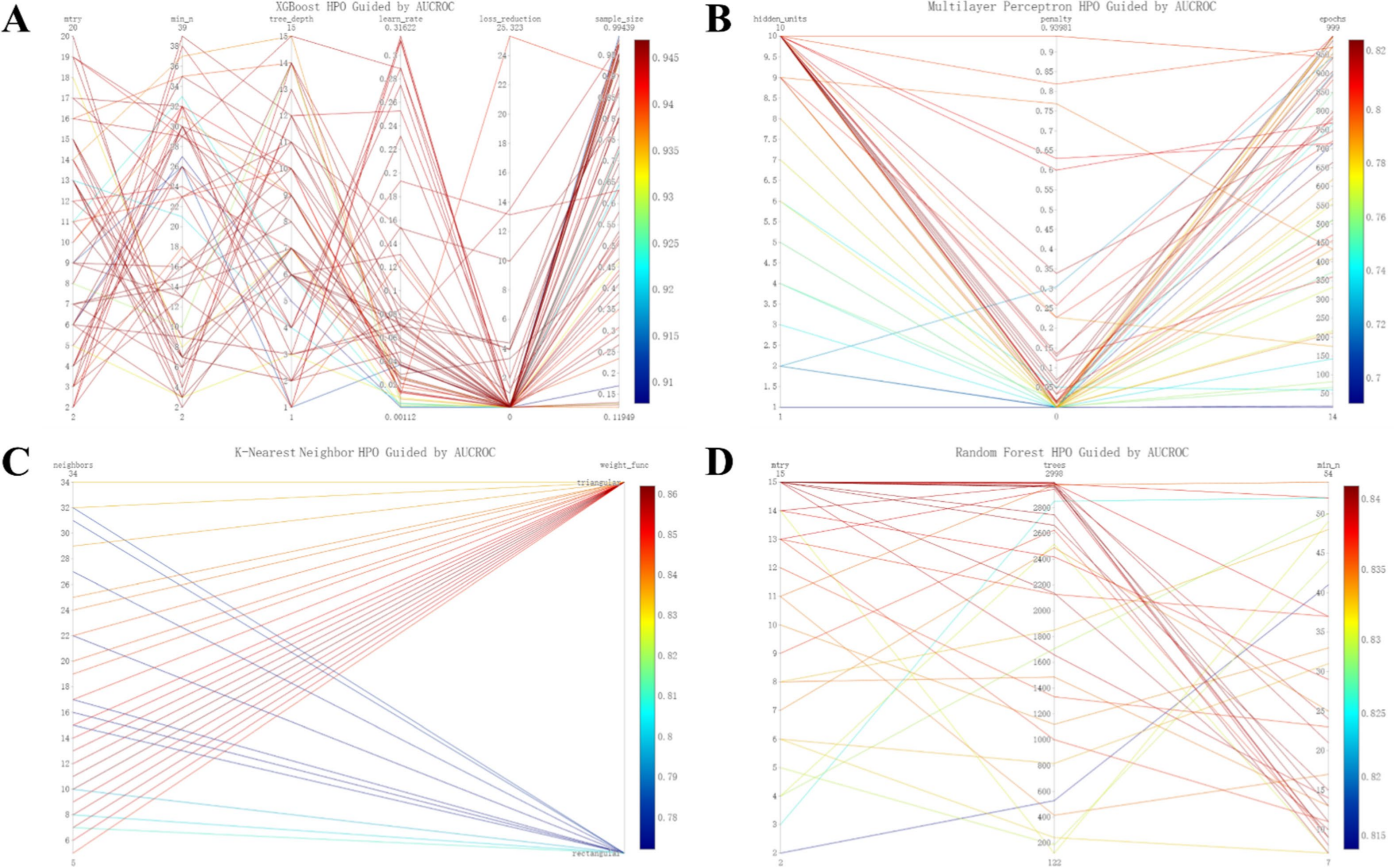
Refined hyperparameter optimization (HPO) process for four distinct machine learning models: XGboost **(A)**, MLP **(B)**, KNN **(C)**, and RF **(D)**.

Figure 3 provides additional insights into the hyperparameter tuning process and the resulting AUCs across the training phase for the machine learning models.

### Assessment on calibration and KS

**Figure 4:**
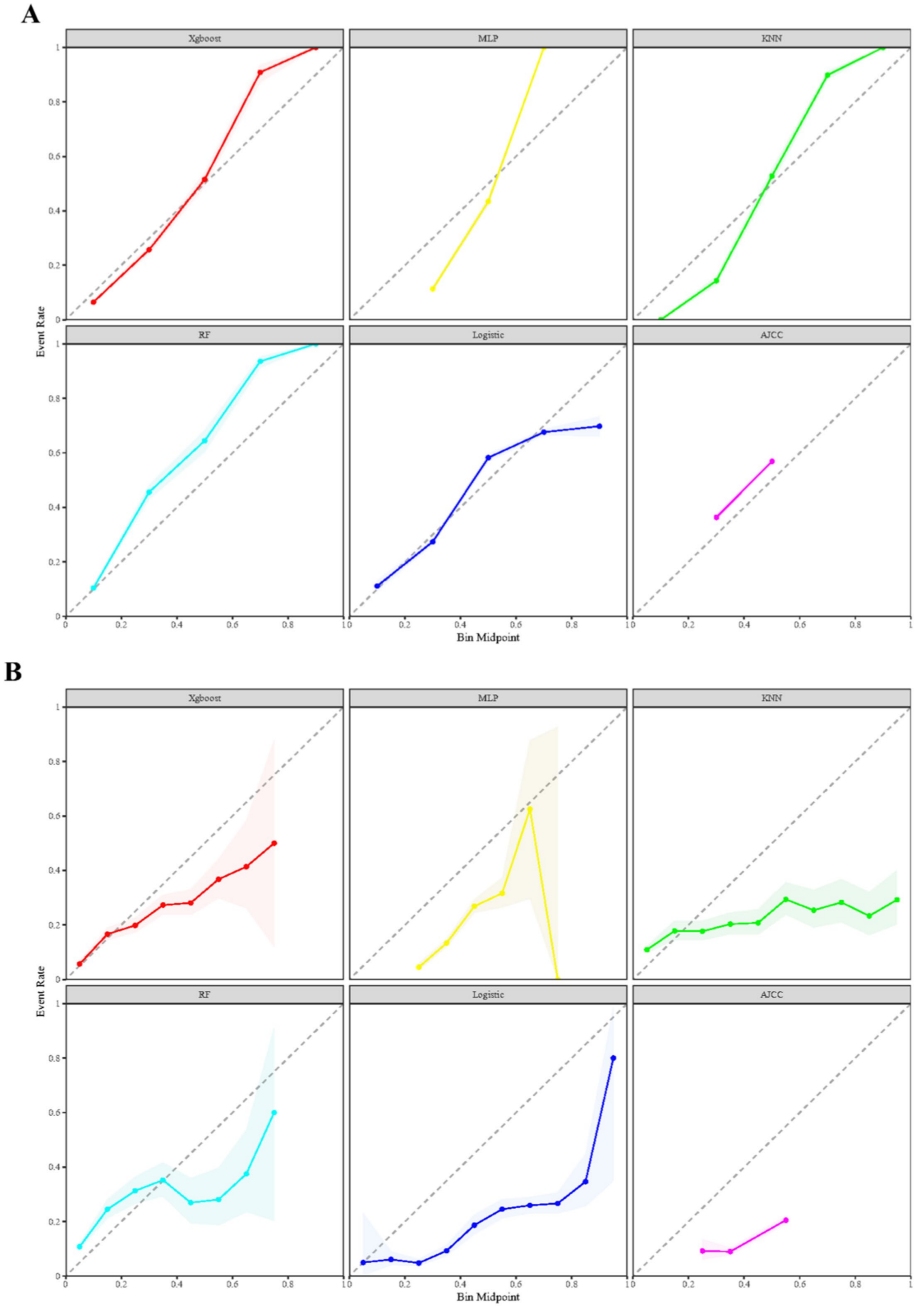
Comparison of prognostic models for calibration curves across both training **(A)** and validation **(B)** datasets.

Figure 4 presents the calibration plots for the training and validation datasets across all constructed models. Notably, the XGBoost model stands out for its superior precision in predicting early mortality, outperforming both machine learning and traditional models in terms of accuracy. This enhanced predictive capability is further supported by the KS statistic in Supplementary Table S1, which show values of 0.802 for the training cohort and 0.666 for the validation cohort. Together, these findings underscore the significant clinical utility of the XGBoost model in accurately prognosticating early mortality for SCLC patients following chemotherapy.

### Model interpretation

**Figure 5:**
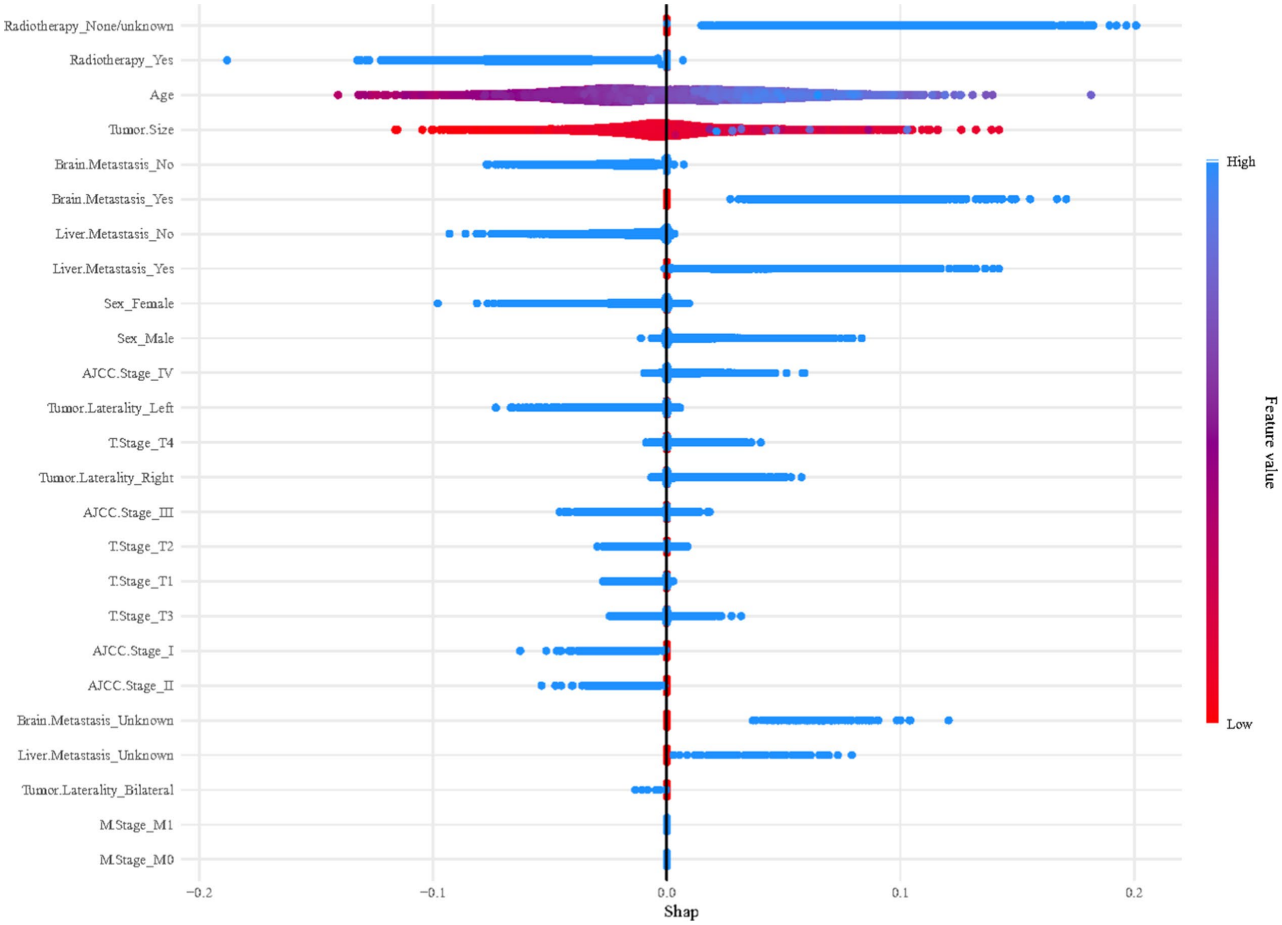
Summary plots of SHAP values in the XGBoost model.

Given the effectiveness of the XGBoost model in forecasting early mortality within the datasets, this study utilized SHAP plots to delineate the feature hierarchy and comprehend their respective impacts on prognosis within the model framework. Figure 5 reveals a distinct pattern, indicating that features with elevated SHAP values are linked to an increased risk of adverse prognosis in SCLC patients post-chemotherapy. The plot’s color spectrum offers further insights, with red signaling smaller eigenvalues, purple for eigenvalues near the mean, and blue for larger eigenvalues. Notably, this analysis highlights the profound impact of radiotherapy and age on mortality risk, with tumor size and metastases to the brain and liver also being significant features.

**Figure 6:**
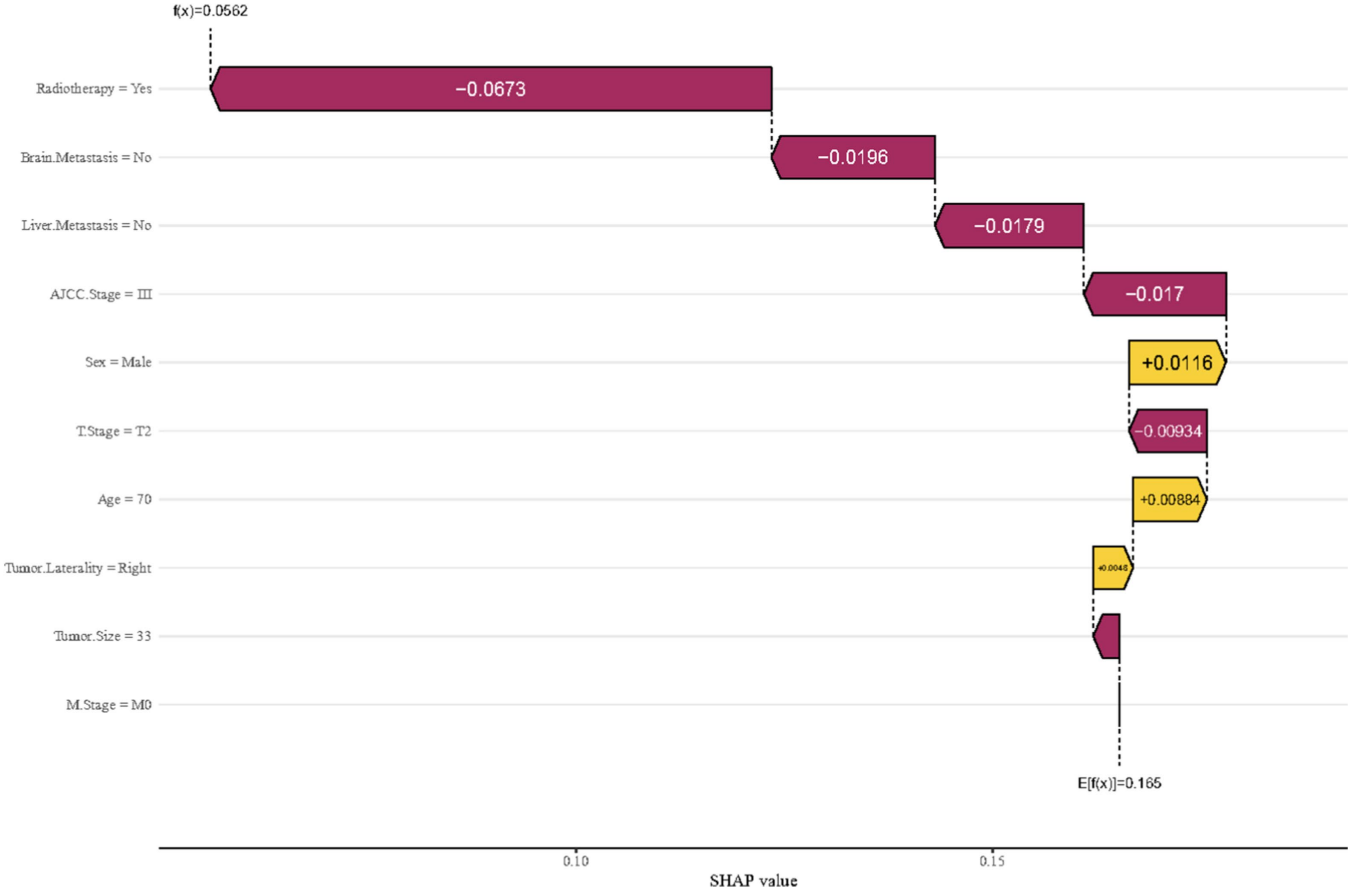
Breakdown analysis utilizing features derived from the first member of the cohort in the XGBoost model.

To delve deeper, this study performed a breakdown analysis on the first individual in the cohort to assess how various features influence the predicted outcome. As illustrated in Figure 6, the model’s initial average predictive value was 0.056. Several features were found to negatively impact the model’s prediction, including radiotherapy, the absence of metastases to the brain and liver, a tumor stage of T2, smaller tumor size, and an AJCC stage of III. On the other hand, features such as being male and having a tumor in the right lung emerged as positive influencers. This analysis ultimately resulted in a final predicted odds of 0.165 for early mortality. The goal of this analysis is to highlight and clarify the key features that contribute to prognostic outcomes in the studied population.

**Figure 7:**
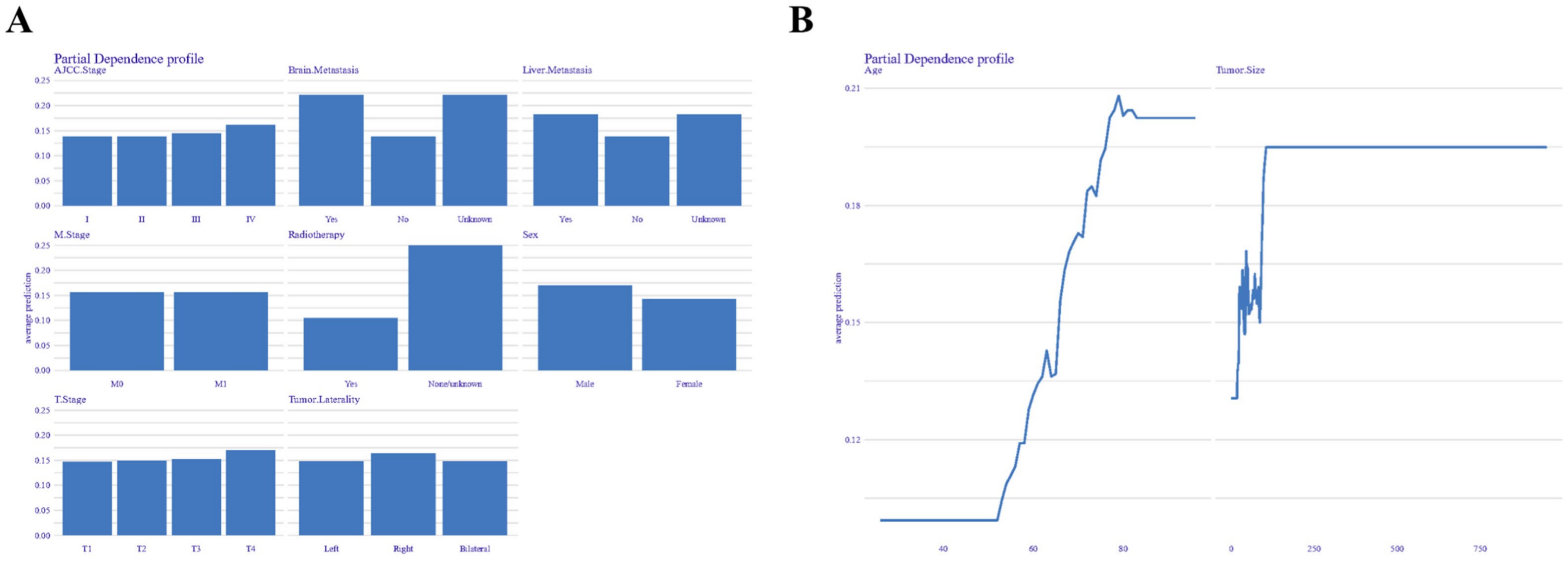
Partial dependence profiles showing the influence of categorical **(A)** and continuous **(B)** features on model performance.

Figure 7 presents the partial dependence profiles, which provide insights into how both categorical (Figure 7A) and continuous (Figure 7B) features influence the model’s performance. By isolating the effects of individual features, the analysis reveals that radiotherapy and brain metastasis have a significant impact on the model’s predictive capabilities. In contrast, factors such as age over 60 and tumor size greater than 250 millimeters are key determinants in shaping the model’s performance.

### Development of a predictive system on a web server

Building on the robust XGBoost model, this study has developed a web-based application that simplifies the predictive assessment of early mortality using patient data as input. This platform is tailored to support researchers without a background in machine learning by providing an intuitive, automated system for setting up, training, and evaluating a XGBoost model. The application’s user-friendly interface is detailed in Supplementary Figure S2 and can be accessed through the following link: https://the-lungcare-innovators-research-team.shinyapps.io/early-mortality-predictor/. It stands as a practical tool for researchers conducting prognostic studies on post-chemotherapy survival in SCLC patients.

## Discussion

This research analyzed 12,500 patients who underwent chemotherapy for SCLC. The XGBoost model outperformed other machine learning models, including MLP, KNN, and RF, as well as traditional methods like logistic regression and AJCC staging in predicting early mortality. A beeswarm summary plot revealed that radiotherapy was the most significant risk factor, followed by age, tumor size, and the presence of brain and liver metastases, highlighting key prognostic indicators for this patient group. The refined XGBoost model was used to develop a web-based prognostic tool, aimed at providing clinicians with personalized insights and potentially transforming patient care. This research represents an innovative approach in applying a machine learning-driven prognostic model specifically for SCLC patients post-chemotherapy, addressing a critical gap in the existing literature.

While recent studies have highlighted the potential of immunotherapy in treating SCLC, there are inconsistencies in their findings. The CASPIAN phase III study found that adding durvalumab, a PDL1 inhibitor, to chemotherapy enhances outcomes in ES-SCLC patients (13). The IMpower133 study echoed these benefits with the addition of atezolizumab (14). Conversely, the CheckMate 331 phase III study observed no survival benefit with nivolumab as a second-line treatment post-chemotherapy (15). Despite new options, chemotherapy remains the unwavering primary treatment for SCLC. Yet, significant early mortality persists post-chemotherapy due to factors like advanced disease stage, metastatic potential, and poor overall health. Therefore, developing a predictive model for this patient group could revolutionize care by enabling personalized treatment plans, improving outcome predictions, and enhancing overall patient survival.

The AJCC and Veterans Administration Lung Study Group (VALSG) staging systems are commonly used for predicting SCLC prognosis but fall short in accurately reflecting the disease’s metastatic potential and treatment response variability. These systems focus mainly on the anatomical extent of tumors, neglecting key biological and molecular factors that could impact prognosis and treatment effectiveness (16, 17). This oversight highlights the need for more nuanced predictive tools that incorporate a broader range of clinical data for better precision in SCLC prognosis. Machine learning offers a promising approach by analyzing diverse data types, such as clinical, genetic, and imaging information, to identify intricate patterns. This enables more precise prognosis predictions, tailored treatment plans, and the discovery of new therapeutic targets, thereby improving patient care and advancing cancer research (18). Nonetheless, there remains a notable gap in developing models specifically designed for predicting survival outcomes in SCLC patients following chemotherapy, regardless of the sophistication of the methods used, be it advanced machine learning techniques or traditional algorithms.

Numerous studies have explored the risk of early mortality in individuals with SCLC. For example, Li et al. (19) discovered that factors like age, sex, clinical stage, the presence of metastases (specifically in the liver and lung), and the absence of treatments (including surgery, radiotherapy, and chemotherapy) significantly impact patients’ prognosis, with their model achieving an AUC of 0.86. Similarly, Chen et al. (9) focused on six clinical parameters that predict early mortality, such as age, advanced AJCC stage, brain metastasis, and the lack of surgical, chemotherapeutic, and radiotherapeutic interventions, achieving an AUC of 0.823. While these models are robust, they tend to apply to the broader SCLC population rather than providing personalized insights for individuals based on specific treatments received.

In the realm of predictive modeling for SCLC post-chemotherapy, Hai et al. (8) identified race, advanced age, higher T stage, multiple organ metastases, and the absence of radiotherapy as key prognostic features for early mortality, though their model showed limited discriminatory power with an AUC of 0.653. Jones et al. (7). found PS and tumor stage to be linked with early mortality, while Lassen et al. (20) pinpointed age, PS, and lactate dehydrogenase levels as predictors for early non-toxic death. Both studies, however, did not culminate in a predictive model, likely due to a paucity of data features. In contrast, this model is specifically designed for SCLC patients post-chemotherapy, providing enhanced precision in predicting early mortality. Utilizing a wide geographical dataset and robust machine learning techniques, this model promises wider applicability for future prognostic assessments.

The prognostic evaluation of SCLC patients revealed that the Lasso algorithm identified 10 independent predictive features, aligning with findings from previous studies (9, 21). Additionally, there is evidence that surgery could affect SCLC outcomes (21, 22). However, due to the retrospective design of the study and the treatment differences between Limited Stage (LS) and ES-SCLC, this study excluded surgical data to avoid potential selection bias. Consequently, this may have led to an underestimation of surgery’s impact in LS-SCLC patients. Therefore, more in-depth studies with prospective design are required to validate and further expand the findings. Furthermore, biomarkers like serum YKL-40 and urea have been associated with early mortality in SCLC (23, 24), but their absence in the database limits the understanding of their roles in this context. This omission could affect the model’s predictive accuracy.

To ensure the robustness of the model, 10-fold cross-validation was utilized to address overfitting and test its generalizability across varied patient groups. Calibration curves confirmed the reliability of the XGBoost model, closely aligning predicted and observed survival probabilities. The model’s validity is further supported by a strong KS score, outperforming other models. Clinical utility was demonstrated by employing DCA, which revealed that the model surpassed conventional ones in predicting net benefits for both the training and validation datasets. These results underscore the model’s potential for clinical adoption, suggesting it could significantly refine decision-making and enhance patient care.

The study’s development of an XGBoost model for a specific patient subset offers promising predictive capabilities; yet, potential limitations must be considered. The retrospective design may introduce selection bias, and while the SEER database is extensive, it lacks detailed information on smoking status, specific treatments, and critical patient characteristics such as genetic profiles, lab results, and comorbidities. Furthermore, despite the use of Lasso regularization and 10-fold cross-validation to minimize overfitting, there is a necessity for external validation to confirm the model’s effectiveness across different datasets and enhance its generalizability.

## Conclusion

This study marks a groundbreaking application of XGBoost models for prognostic evaluation in post-chemotherapy SCLC patients. The innovative predictive tool empowers clinicians to customize treatment plans based on individual patient profiles. Additionally, it aids in the strategic planning of follow-up appointments, thereby refining and personalizing the approach to patient care.

## Data Availability

Publicly available datasets were analyzed in this study. This data can be found here: https://seer.cancer.gov/.

## Footnotes

^1^Accessible at https://seer.cancer.gov/data-software/.

^2^ https://www.r-project.org/
