# Supplementary material for: Machine learning insights into early mortality risks for small cell lung cancer patients post-chemotherapy
Source: Front Med (Lausanne). 2025 Jan 24;12:1483097. doi: 10.3389/fmed.2025.1483097 (PMC11802579; doi:10.3389/fmed.2025.1483097)
Supplement: Supplementary file 1 [file Supplementary_file_1.docx]

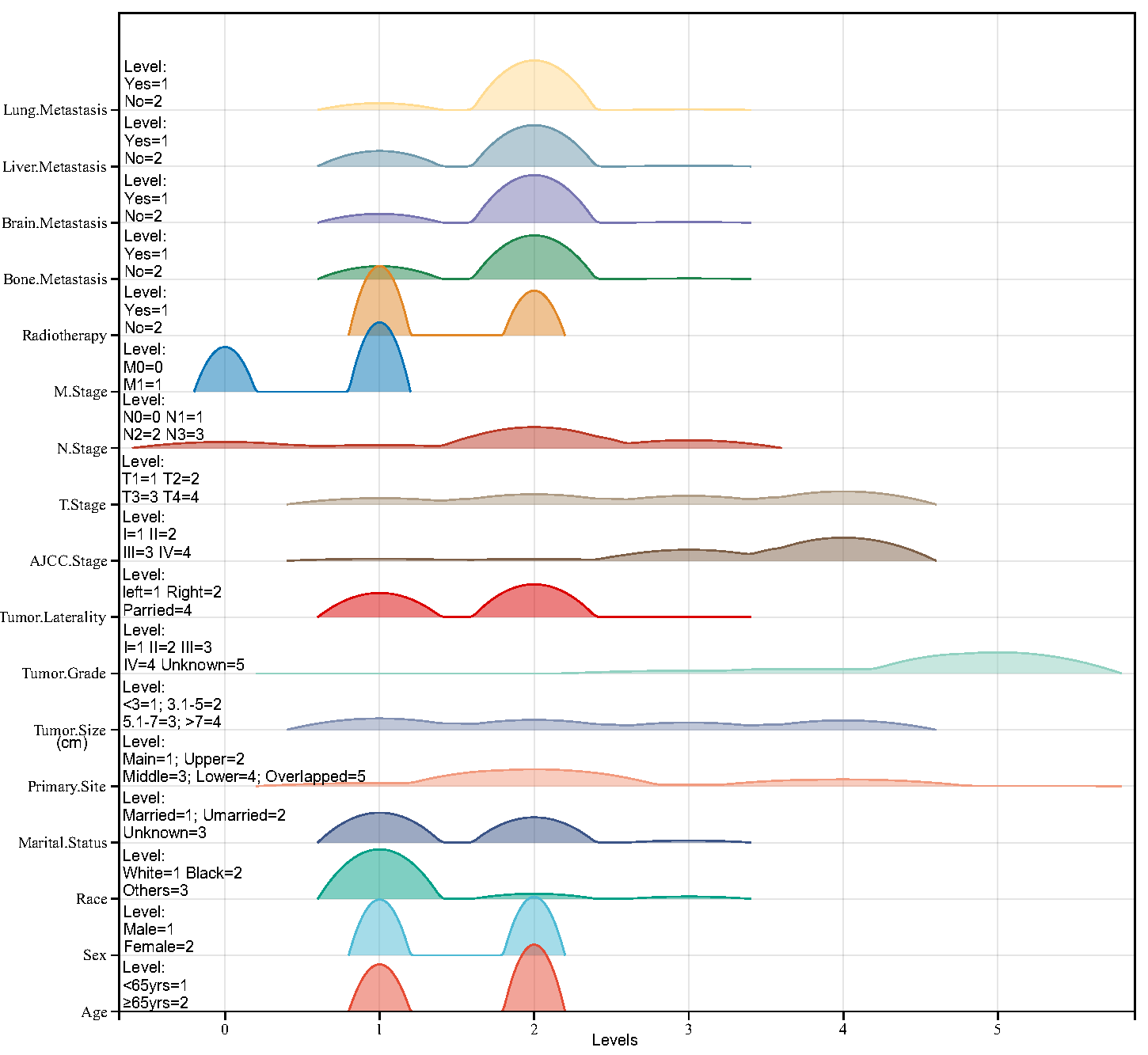


**Supplementary Figure 1**: Ridge plot illustrating the distribution of features across the dataset.
