# Supplementary figures and images for: Machine learning insights into early mortality risks for small cell lung cancer patients post-chemotherapy
Source: Front Med (Lausanne). 2025 Jan 24;12:1483097. doi: 10.3389/fmed.2025.1483097 (PMC11802579; doi:10.3389/fmed.2025.1483097)

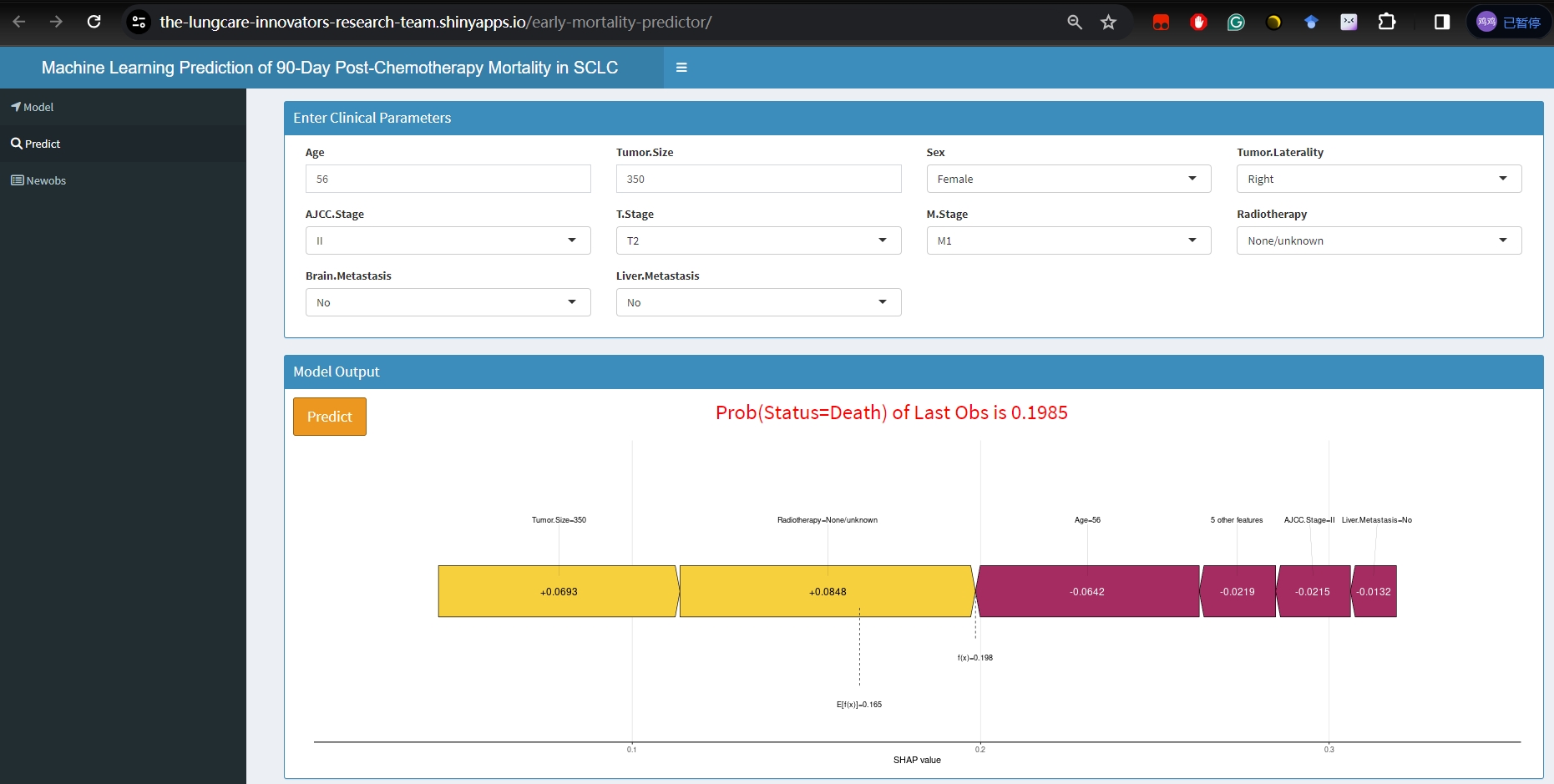


**Supplementary Figure 2.** Online web server interface for the XGBoost model.

Supplement: Supplementary file 2 [file Supplementary_file_2.docx]
