# Supplementary material for: Machine learning insights into early mortality risks for small cell lung cancer patients post-chemotherapy
Source: Front Med (Lausanne). 2025 Jan 24;12:1483097. doi: 10.3389/fmed.2025.1483097 (PMC11802579; doi:10.3389/fmed.2025.1483097)
Supplement: Supplementary file 3 [file Table_1.docx]

Supplementary Table 1. Comparison of the Kolmogorov-Smirnov statistic among the six models in the training and validation datasets.

| Model | Kolmogorov-Smirnov statistic | |
| --- | --- | --- |
|  | Training set | Validation set |
| XGBoost | 0.802 | 0.666 |
| Multilayer Perceptron | 0.513 | 0.321 |
| K-Nearest Neighbor | 0.571 | 0.365 |
| Random Forest | 0.673 | 0.398 |
| Logistic regression | 0.367 | 0.347 |
| AJCC staging | 0.205 | 0.183 |
